# Vibration therapy to improve pain and function in patients with chronic low back pain: a systematic review and meta-analysis

**DOI:** 10.1186/s13018-023-04217-2

**Published:** 2023-09-26

**Authors:** Qiang Li, Pan Liu, Zongbao Wang, Xin Li

**Affiliations:** 1 School of Acupuncture and Massage, Anhui University of Chinese Medicine, No.103 Meishan Road, Shushan District, Hefei, 230038 Anhui People’s Republic of China; 2 Anhui Provincial Hospital of Integrated Chinese and Western Medicine (The Third Affiliated Hospital of Anhui University of Chinese Medicine), No. 45 Shihe Road, Shushan District, Hefei, 230031 Anhui People’s Republic of China; 3 Shuguang Anhui Hospital Affiliated to Shanghai University of Traditional Chinese Medicine, No. 45 Shihe Road, Shushan District, Hefei, 230031 Anhui People’s Republic of China

**Keywords:** Vibration therapy, Chronic low back pain, Randomized controlled trial

## Abstract

**Background:**

Vibration therapy (VT), a treatment of musculoskeletal disorders, has been developed for clinical applications in the past decade. However, its effect on relieving chronic low back pain (CLBP) and improving lumbar function is still illusive, lacking sufficient evidence-based medical data.

**Objective:**

This systematic review aimed to evaluate the efficacy of vibration therapy on pain and function in people with CLBP.

**Methods:**

PubMed, Cochrane Library, Web of Science, Embase, CNKI, Wanfang Date, VIP, and CBM were applied to search for clinical randomized controlled trials (RCTs) on vibration therapy for people with CLBP. The electronic databases were searched from the establishment of the database until July 1, 2023. Two researchers assessed the quality of the included studies and extracted data. The outcome indicators included the pain intensity index, Oswestry dysfunction index (ODI) score, and Roland–Morris dysfunction questionnaire (RMDQ) score. GRADE was used to evaluate the certainty of evidence of each outcome indicator. The meta-analysis was conducted using RevMan 5.3 software.

**Results:**

Fourteen papers met the inclusion criteria with 860 subjects (VT group *n* = 432 and control group *n* = 428). VT for patients with CLBP reduced the pain intensity index [SMD = − 0.71, 95% CI (− 1.02, − 0.39), *I*^2^ = 76%, *P* < 0.0001], the ODI score value [MD = − 4.24, 95% CI (− 8.10, − 0.38), *I*^2^ = 88%, *P* = 0.03], and the RMDQ score value [MD = − 2.21, 95% CI (− 3.41, − 1.01), *I*^2^ = 0%, *P* = 0.0003]. Subgroup analysis displayed that the pain intensity index was lower in the whole-body vibration (WBV) group than in the control group [SMD = − 0.49, 95% CI (− 0.79, − 0.19), *I*^2^ = 58%, *P* = 0.001] and the local vibration (LV) group [SMD = − 1.07, 95% CI (− 1.60, − 0.53), *I*^2^ = 76%, *P* < 0.0001]. The ODI scores in the WBV group were lower than those in the control group [MD = − 3.30, 95% CI (− 5.76, − 0.83), *I*^2^ = 36%, *P* = 0.009]. There was no statistically significant difference in ODI scores between the LV group and the control group [MD = − 5.78, 95% CI (− 16.23, 4.66), *I*^2^ = 97%, *P* = 0.28].

**Conclusion:**

The data from this study suggest that VT can reduce pain and improve lumbar function in patients with CLBP. However, we still need to carefully interpret the results of this study, as the certainty of evidence was low, and the clinical relevance of the results is questionable. Further RCTs are needed in the future to ascertain this.

**Supplementary Information:**

The online version contains supplementary material available at 10.1186/s13018-023-04217-2.

## Introduction

Low back pain (LBP), also known as lower back pain, lumbar back pain, etc., is pain arising from the lower border of the ribs, in the lumbosacral and sacroiliac regions, with or without radiating pain to the lower extremities. A duration of more than 3 months of the disease is considered chronic low back pain [1]. A total of 80% of adults experience low back pain [2], and CLBP can incapacitate people and reduce the possibility of early return to work [3]. Treatments such as oral medications and suspension training have limited efficacy in CLBP, which is a common disease with complex etiology and pathogenesis [4]. It can have a serious impact on the health, quality of life, and work of people, as well as bringing about heavy medical costs and indirect social burdens [4].

In the past decade, vibration therapy has been developed in clinical applications, but has not yet been widely applied in the rehabilitation of CLBP. Vibration therapy has the advantages of safety and saving manpower and material resources. Especially in the field of geriatric rehabilitation, it has been proven to have a high degree of safety, and there has been no report of serious adverse reactions to vibration [5]. Vibration therapy includes whole-body vibration therapy and local vibration therapy. The previous systematic reviews and meta-analyses of vibration therapy for CLBP [6, 7] are few and only focused on whole-body vibration therapy on CLBP. As the clinical use of vibration therapy for low back pain continues to increase, there is a need to include local vibration therapy in evidence syntheses including meta-analysis.

To this end, we performed a meta-analysis of RCTs of not only whole-body vibration therapy but also local vibration therapy of people with CLBP. The purpose of this study is to comprehensively analyze the effects of vibration therapy on pain and function in patients with CLBP and to provide further clinical data-based evidence for the treatment of CLBP.

## Methods

### Study design

The meta-analysis was registered on the PROSPERO platform of the International Register of Systematic Evaluations (No. CRD42023429930). It is in accordance with the guidelines of Preferred Reporting Items for Systematic Reviews and Meta-Analyses [8, 9] and Cochrane Handbook [10].

### Search strategy

Randomized controlled trials of vibration therapy for CLBP were searched in the English databases PubMed, Cochrane Library, Web of Science, Embase, and in the Chinese databases CNKI, Wanfang Date, VIP, and CBM. The electronic databases were searched from the establishment of the database until July 1, 2023, using Mesh Terms, index terms, and keywords. The search strategy was developed according to the Patient population, Intervention, Comparison, Outcome, and Study design (PICOS) approach. Chinese search terms: "vibration" or "whole-body vibration" or "vibration therapy" and "low back pain" or "chronic low back pain" or "lumbar muscle strain" or "lumbar paralysis" and "randomized controlled trial" or "randomized controlled study" or "RCT" or "randomized." The English search strategy is shown as an example in the Additional file 1.

### Inclusion criteria

➀ Study type: RCTs.
➁ Literature language: Chinese and English.
➂ Study subjects: Patients with CLBP (pain arising from the lower edge of the ribs, in the lumbosacral and sacroiliac areas, with or without radiating pain in the lower limbs, and the duration of the disease is more than 3 months) for all races, nationalities, and duration of the disease.
➃ Intervention methods: The vibration therapy or vibration therapy combined with additional treatment, including basic medication, exercise therapy, and others, was applied in the VT group. Interventions other than vibration therapy were applied in the control group.
➄ Outcome indicators: Pain intensity indicators (visual analog scale (VAS) and numeric rating scales (NRS)) and functional indicators (Oswestry disability index (ODI) and Roland–Morris disability questionnaire (RMDQ)).

### Exclusion criteria

(1) The literature with missing data; (2) the literature without complete text content and duplicate articles; (3) non-RCT studies; (4) the literature with non-compliant research content; (5) the literature with different research subjects, research methods, and outcome indicators; (6) dissertations and low-quality literature; and (7) the literature for patients with lumbar radiculopathy or neural problems.

### Screening and data extraction

Two researchers (QL and PL), both with search experience and training, read the literature to screen and extract information based on inclusion and exclusion criteria. The data recorded included: author, publication time, country, number of people, age, intervention, duration of intervention, and outcome indicators. Two researchers (QL and PL) checked the results of the collected data from each other, and disagreements were discussed between researchers to reach a consensus. The corresponding author (ZBW) made the final decision if disagreements persisted.

### Risk of bias evaluation

Two researchers (QL and PL) assessed the quality of the included studies, using the Cochrane Handbook's Risk of Bias Assessment Tool for RCTs [11]. Any disagreements were resolved by reaching an agreement through joint discussion.

### Certainty of evidence assessment

We used the Grading of Recommendations, Assessment, Development, and Evaluation (GRADE) system [12] to classify the certainty of evidence for outcome indicators of the included studies. And five factors in the system could affect the certainty of evidence. The grade of evidence is categorized as high, medium, low, and very low.

### Statistical analysis

RevMan 5.3 software was used in the data analysis process. Since the data are a continuous variable, we utilize the mean difference (MD) and standard mean difference (SMD) as impact indicators to provide a 95% confidence interval (CI). The statistical heterogeneity among the studies in each meta-analysis was tested using the *I*^2^ test and the *Chi*^2^ test. At *P* ≥ 0.1 and *I*^2^ ≤ 50%, there was no significant heterogeneity in the included literature, which could be analyzed by meta-analysis using the fixed-effects model. In the case of *P* < 0.1, *I*^2^ > 50%, there was significant heterogeneity in the included literature, and meta-analysis could be performed to use the random effects model. To identify sources of heterogeneity, subgroup analysis was conducted based on vibration therapy modality. In addition, we assessed the publication bias among the included studies by the funnel plot and Egger's test.

## Results

**Fig. 1:**
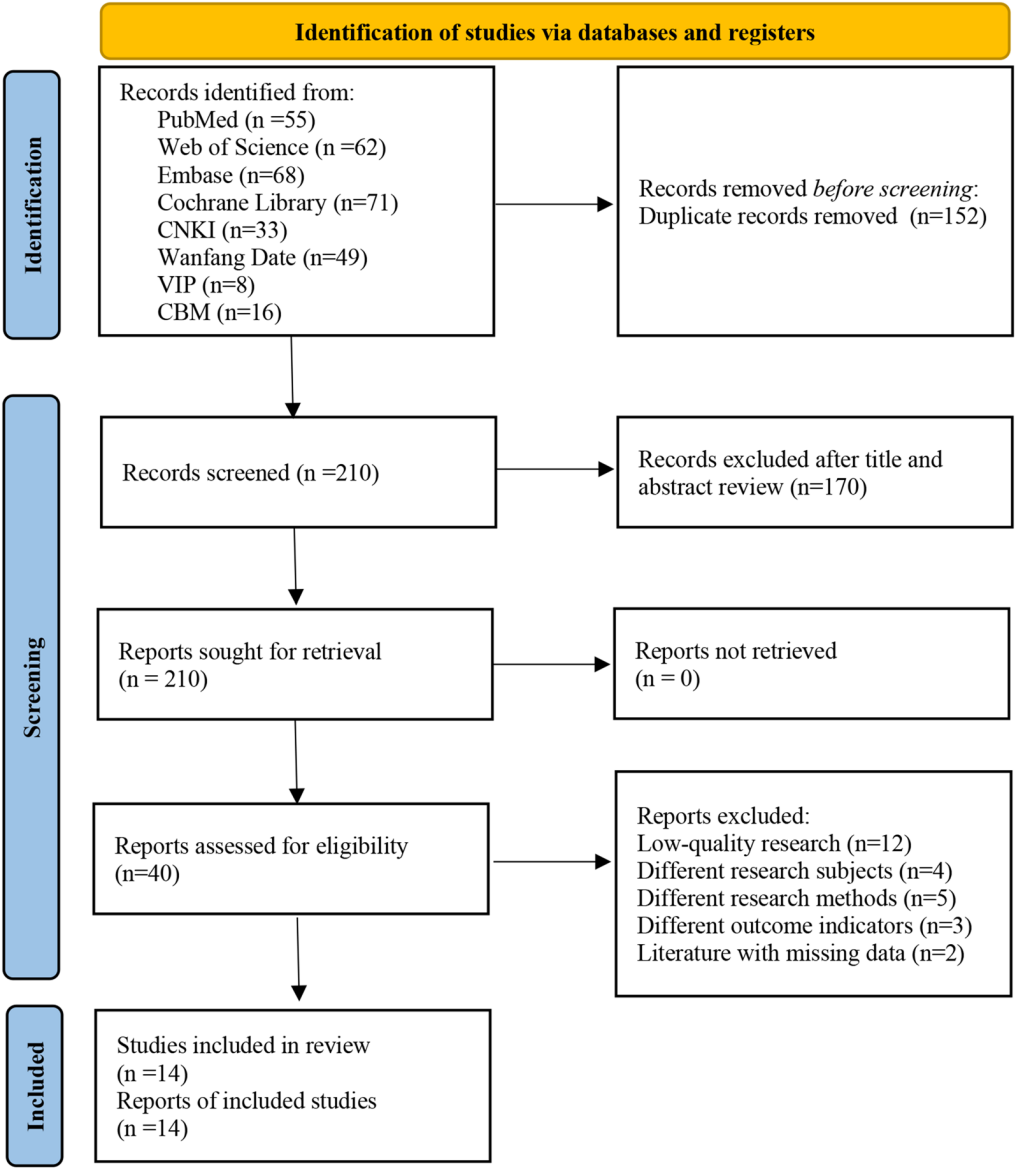
PRISMA flowchart of studies screening selection

The initial literature search of the database retrieved a total of 362 records. A total of 152 repetitive records were detected and removed using the EndNote X9 software. In addition, after reviewing the title and abstract, 170 records were not included, with a Kappa score of 0.84 (95% CI 0.83–0.90). After full-text reading, 26 studies did not match the inclusion requirements, with a Kappa score of 0.89 (95% CI 0.79–0.99). Finally, this study included 14 RCTs [13–26] with a total of 860 people (*n* = 432 in the VT group and *n* = 428 in the control group). Figure 1 shows the PRISMA flowchart of studies screening selection.

### Basic characteristics of included studies and risk of bias evaluation

Fourteen papers were published between 2002 and 2023 from China, Spain, Korea, Germany, and Turkey. In the experimental group, all individuals received vibration therapy or vibration therapy in combination with other treatment regimens, while the control group received interventions other than vibration therapy. The duration of the interventions varied from 2 to 24 weeks. Table 1 shows the basic features of the 14 RCTs.

**Table 1:** Basic characteristics of the included studies

References	Country	Number of patients T/C	Average age (years)		Interventions		Outcome indicators	Intervention time (weeks)
			T	C	T	C		
Chen [13]	China	20/20	66.4 ± 9.9	67.6 ± 8.2	WBV	Oral medication	(1)	24
Zhang [14]	China	30/30	37.93 ± 4.14	38.07 ± 4.24	Control group + LV	Core training, stereo-interferential electrotherapy, and health promotion	(1)	4
Yuan [15]	China	40/41	37.67 ± 11.41	36.73 ± 11.72	Control group + LV	Chinese herbal fumigation and ultrashort wave therapy	(1)(2)	2
Chen [16]	China	51/51	52.13 ± 7.35	53.73 ± 7.78	Control group + LV	Lumbar spine training and oral medication	(1)(2)	24
del Pozo-Cruz [17]	Spain	25/24	58.71 ± 4.59	59.53 ± 5.47	WBV	Daily activities	(1)(2)(3)	12
Wang [18]	China	45/44	21.64 ± 3.01	22.02 ± 4.59	Control group + WBV	Exercise routines	(1)(2)	12
Jung [19]	Korea	25/25	18.00 ± 0.6	18.04 ± 0.68	Control group + WBV	General trunk movements	(1)	12
Rittweger [20]	Germany	25/25	54.1 ± 3.4	49.8 ± 6.6	WBV	Lumbar spine stretching exercises	(1)	12
Kaeding [21]	Germany	21/20	46.4 ± 9.3	44.6 ± 9.1	WBV	Daily activities	(2)(3)	12
Kim [22]	Korea	18/18	46.81 ± 10.6	42.09 ± 7.4	Control group + LV	Suspension training	(1)(2)	4
Karacay [23]	Turkey	25/24	43.3 ± 9.2	43.6 ± 9.4	WBV	Regular exercise plan	(3)	8
Micke [24]	Germany	70/70	54.3 ± 7.8	58.3 ± 7.5	WBV	Regular exercise plan	(1)	12
Yang [25]	Korea	20/20	32.8	30.95	Control group + WBV	Lumbar stability training	(1)(2)	6
Wegener [26]	Germany	17/16	60.9 ± 8.2	63.9 ± 6.5	WBV	Classic physiotherapy	(2)	6

(1) Pain intensity indicators: visual analog scale (VAS) and numeric rating scales (NRS)

(2) Oswestry disability index (ODI)

(3) Roland–Morris disability questionnaire (RMDQ)

**Fig. 2:**
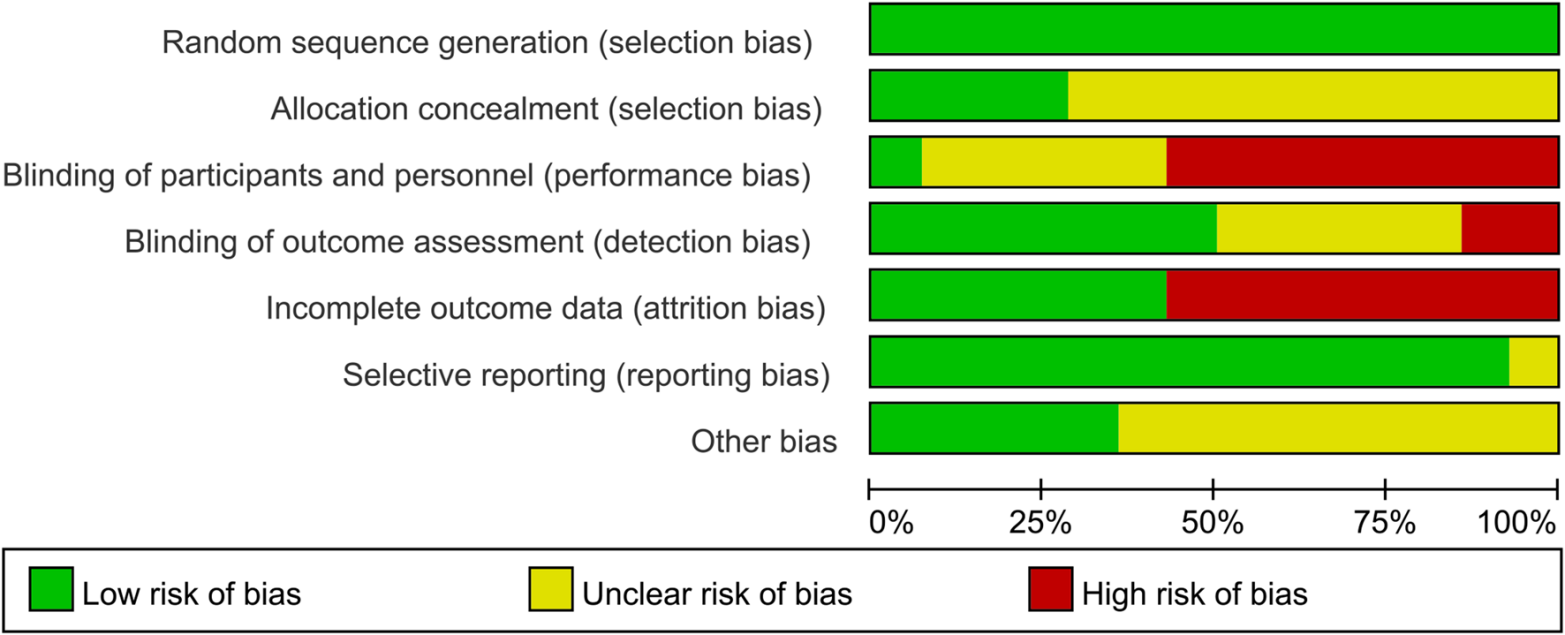
Risk of bias graph

**Fig. 3:**
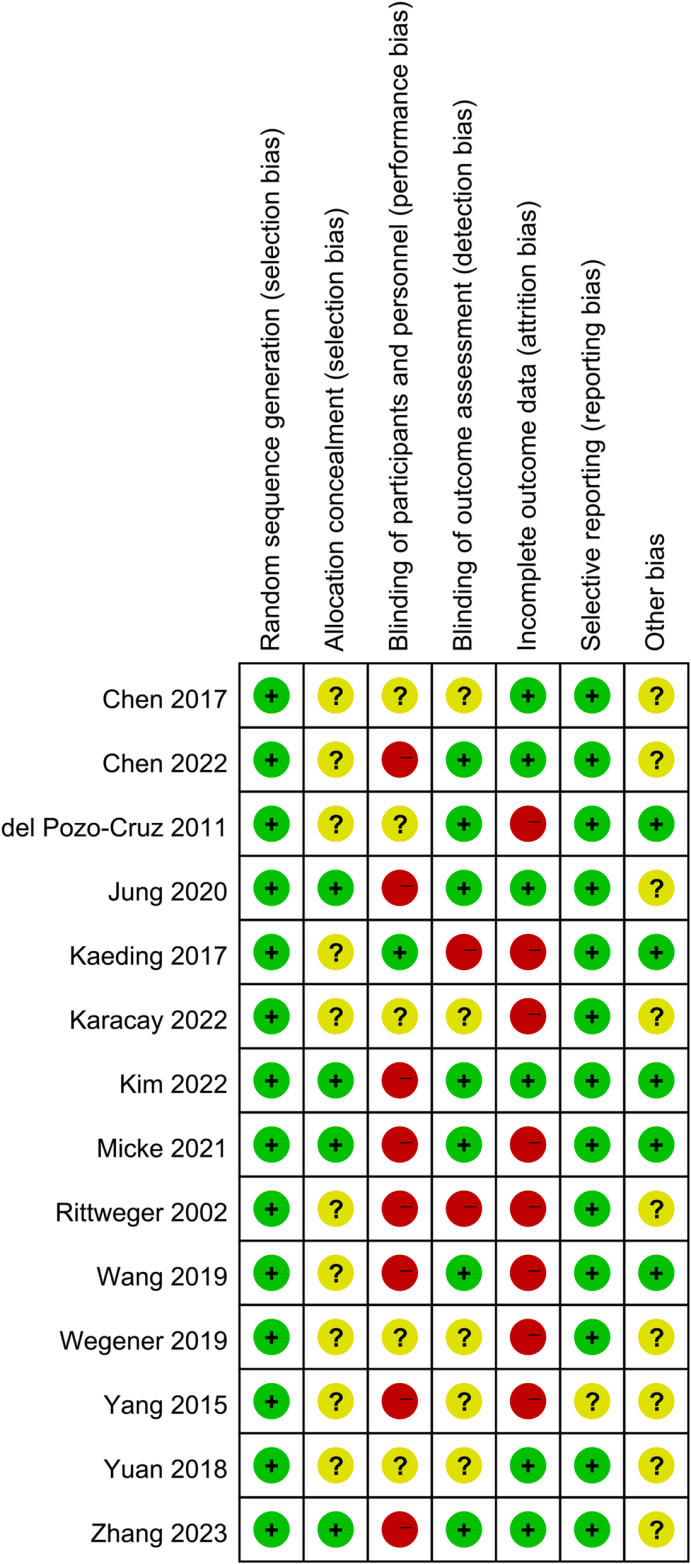
Risk of bias summary

All studies explicitly used random grouping. Four studies [14, 19, 22, 24] described allocation concealment. One study [21] used a blind method for participants, and seven studies [14, 16–19, 22, 24] used blind methods for evaluators. The evaluation results of the 14 RCTs are shown in Figs. 2 and 3.

### Assessment of certainty of evidence

The included outcome indicators were assessed based on GRADE, which showed a low certainty of evidence for the pain intensity index, the ODI score, and the RMDQ score. For more detailed information, please refer to Additional file 2: Table S1.

### Meta-analysis results

The interventions in 14 RCTs were mainly categorized into whole-body and local vibration therapy, and the outcome indicators included VAS, NRS, ODI, and RMDQ scores. Because the results of the VAS and NRS scores were similar, they were pooled together in the meta-analysis presented in a forest plot. This is a common approach within the Cochrane Back and Neck Group [27].

### Pain scores

**Fig. 4:**
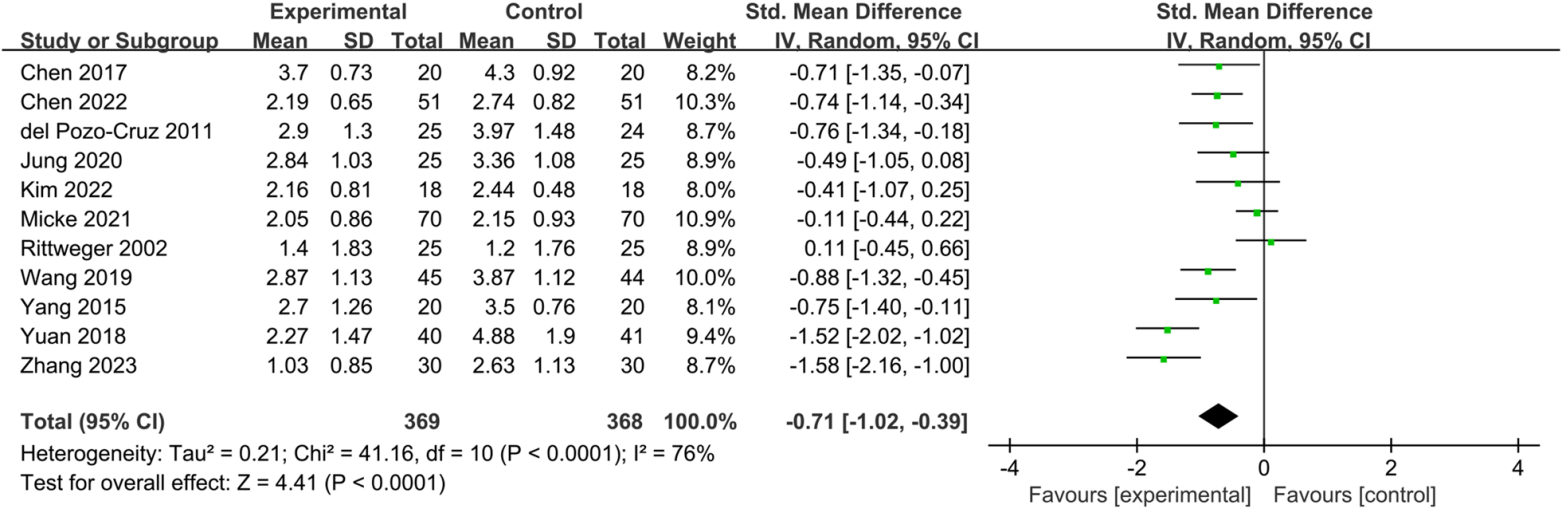
Forest plot of pain scores

Eleven RCTs [13–20, 22, 24, 25] reporting VAS or NRS scores of CLBP people before and after treatment were included in this study. Figure 4 shows the effect of vibration therapy on subjective pain levels in CLBP people, with a total of 737 cases. The results of these 11 studies showed heterogeneity among the results (*I*^2^ = 76%), and the random effects model was chosen to combine them. Meta-analysis showed that, except for Rittweger's study [20], the pain index scores in the VT group were significantly lower than those in the control group [SMD = − 0.71, 95% CI (− 1.02, − 0.39), *I*^2^ = 76%, *P* < 0.0001]. Due to the high spatial requirements for lumbar extension in Rittweger's control group, the use of vibration therapy was more practical.

### ODI scores

**Fig. 5:**
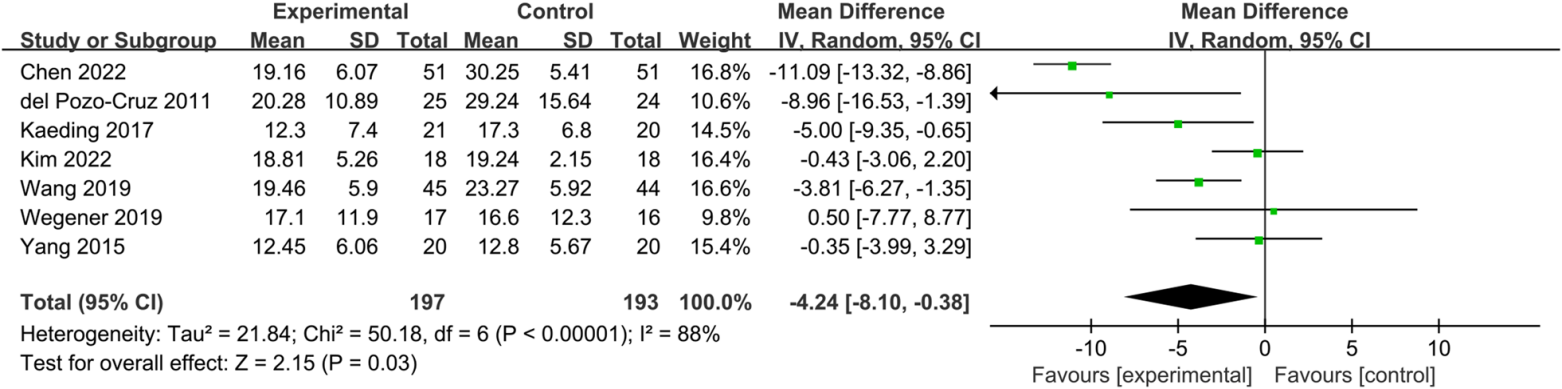
Forest plot of ODI scores

Eight RCTs were included in this study, which reported the ODI scores before and after treatment. However, due to the lack of a sexual function indicator, the ODI of Yuan's study [15] was excluded. Only seven RCTs [16–18, 21, 22, 25, 26] were included for meta-analysis. Figure 5 gives the effect of vibration therapy on ODI scores in CLBP patients with a total of 390 cases. The heterogeneity of the seven studies was considerable (*I*^2^ = 88%); therefore, a random effects model was used for analysis. Meta-analysis showed that ODI scores in the VT group were significantly lower than those in the control group [MD = − 4.24, 95% CI (− 8.10, − 0.38), *I*^2^ = 88%, *P* = 0.03].

### RMDQ scores

**Fig. 6:**
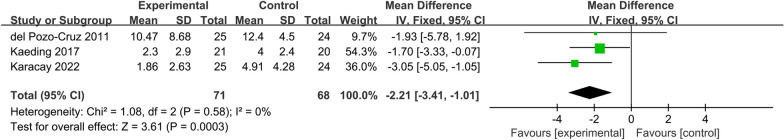
Forest plot of RMDQ scores

Three RCTs [17, 21, 23] reporting RMDQ scores before and after treatment were included in this study. The impact of VT on RMDQ scores in a total of 139 CLBP patients is shown in Fig. 6. There was no heterogeneity in the three RCTs (*I*^2^ = 0%). Therefore, a fixed-effect model was selected for analysis. Meta-analysis showed that the RMDQ scores in the VT group were significantly lower than those in the control group [MD = − 2.21, 95% CI (− 3.41, − 1.01), *I*^2^ = 0%, *P* = 0.0003].

### Subgroup analysis

#### Pain scores

The eleven included RCTs that reported VAS or NRS scores in people with CLBP were separated into two groups according to vibration mode: whole-body vibration group [13, 17–20, 24, 25] and local vibration group [14–16, 22].

This study showed that the pain intensity index of the whole-body vibration group was significantly lower than that of the control group [SMD = − 0.49, 95% CI (− 0.79, − 0.19), *I*^2^ = 58%, *P* = 0.001], with a significant difference.

Heterogeneity among the findings was considerable in the local vibration group (*I*^2^ = 76%). Meta-analysis showed that the pain intensity index was significantly lower in the local vibration group than in the control group [SMD = − 1.07, 95% CI (− 1.60, − 0.53), *I*^2^ = 76%, *P* < 0.0001], and the difference was statistically significant.

**Fig. 7:**
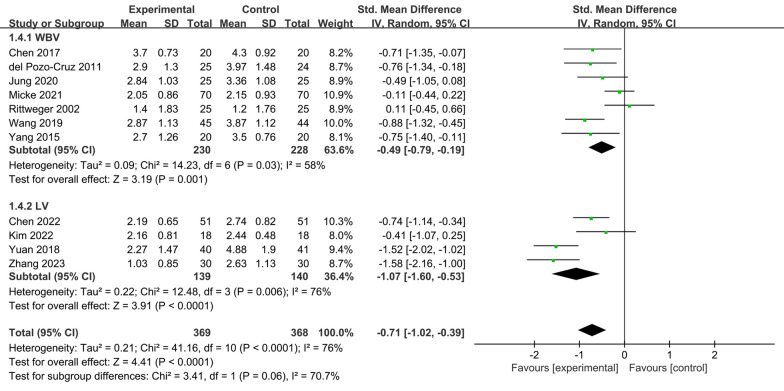
Subgroup analysis of pain scores

We conducted the leave-one-out method on the WBV and LV groups, respectively, to explore the sources of heterogeneity. The results showed that excluding Micke's study [24] in the WBV group reduced heterogeneity to 42% (*P* = 0.0002). However, deleting Yuan's study [15] in the LV group can only reduce *I*^2^ to 75% (*P* < 0.0001) (Fig. 7).

#### ODI scores

The seven included RCTs that reported ODI scores in CLBP patients were separated into two groups according to vibration mode: whole-body vibration group [17, 18, 21, 25, 26] and local vibration group [16, 22].

The heterogeneity among the findings was moderate in the whole-body vibration group (*I*^2^ = 36%). This study showed that the ODI scores in the whole-body vibration group were significantly lower than that in the control group [MD = − 3.30, 95% CI (− 5.76, − 0.83), *I*^2^ = 36%, *P* = 0.009], and the difference was statistically significant.

**Fig. 8:**
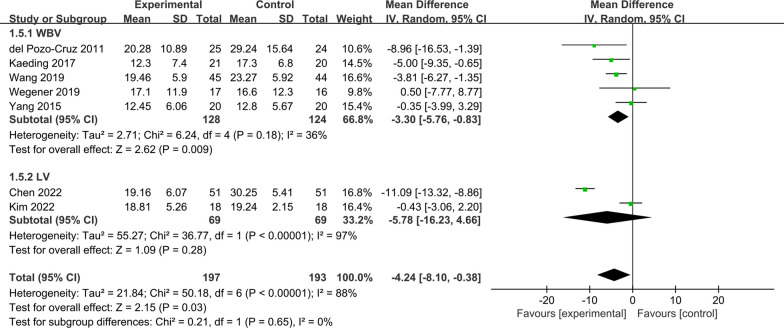
Subgroup analysis of ODI scores

Heterogeneity among the findings was considerable in the local vibration group (*I*^2^ = 97%), and meta-analysis showed that there was no statistically significant difference in the ODI scores of the local vibration group compared with the control group [MD = − 5.78, 95% CI (− 16.23,4.66), *I*^2^ = 97%, *P* = 0.28]. This suggests that local vibration therapy may not be able to significantly improve lumbar function in patients with CLBP (Fig. 8).

#### Publication bias

**Fig. 9:**
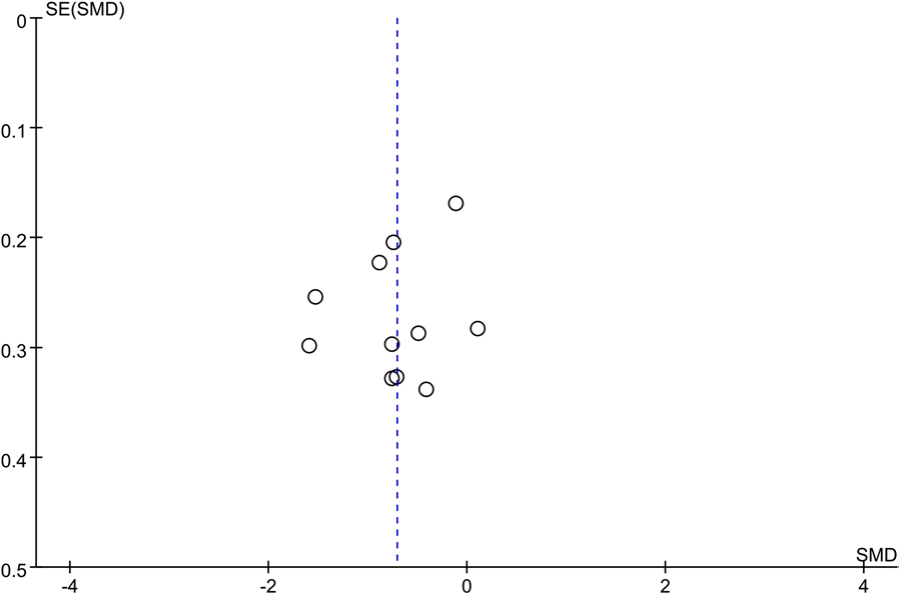
Funnel plot of pain scores

We assessed the publication bias among the included studies on the pain intensity index by the funnel plot and Egger's test. The funnel plot showed a basically symmetrical scatter point, with Egger's test* P* = 0.354, indicating no significant publication bias in the included studies. Therefore, the systematic review results were credible (Fig. 9).

## Discussion

LBP is one of the most common health problems among adults. Within 3 months, myorelaxants, nonsteroid anti-inflammatory drugs (NSAIDs), and NSAIDs with paracetamol can effectively reduce pain and disability in patients [28]. After 3 months, it can develop into CLBP. There are various treatment modalities for CLBP at present. The previous studies have shown that baclofen, duloxetine, NSAIDs, opiates, etc., improved symptoms in patients with CLBP [29–31]. Among them, selective NSAIDs are the most effective non-opioid drugs for the treatment of CLBP [32]. In addition, dextrose prolotherapy and acupuncture may also achieve certain therapeutic effects [33, 34]. The literature in this study includes treatment with oral medication, core muscle training, herbal fumigation therapy, ultrashort wave therapy, lumbar spine stretching exercises, and suspension training in the control group. In the VT group that included vibration therapy, patients' VAS, NRS, ODI, and RMDQ scores decreased significantly, and there were no serious complications, which quantitatively proved that vibration therapy can effectively relieve pain and improve lumbar dysfunction in patients with CLBP. In this study, the results of the pain intensity index and ODI score showed heterogeneity. Subsequent subgroup analysis showed that age, duration of intervention, and vibration pattern were not the causes of heterogeneity, and it was speculated that the etiology of heterogeneity may be related to the subjects included in the study, the treatment regimen received, etc. Subgroup analyses were conducted for the pain intensity index and ODI scores within the outcome indicators, with grouping based on both vibration modalities. The results showed that both whole-body vibration therapy and local vibration therapy improved patients' low back pain. In terms of improvement of lumbar dysfunction, the whole-body vibration group was superior to the control group. The difference between the local vibration group and the control group was not statistically significant, which could be related to the relatively small sample sizes in the included RCTs. Compared with local vibration therapy, whole-body vibration therapy has a wider application. Whole-body vibration therapy involves having the subject stand on a platform, vibrate at a predetermined frequency and amplitude, which is subsequently transmitted throughout the body. Whole-body vibration therapy has been used clinically in the rehabilitation of knee osteoarthritis [35], stroke [36], and cerebral palsy [37], among others. Whole-body vibration therapy for CLBP has many advantages. Firstly, some studies [38–41] have shown that WBV can significantly activate trunk muscle fibers and improve trunk muscle strength, which is helpful in the prevention and treatment of CLBP. Secondly, WBV can relax the lower back muscles to relieve pain [42]. Third, WBV can improve proprioceptive function by activating proprioceptors. This leads to better improvement of spinal dysfunction and instability in patients with CLBP [43, 44]. Local vibration can also be beneficial in the treatment of CLBP, including the use of an automated mechanical device [45] to deliver local mechanical vibration directly or indirectly to the muscles or tendons and joints of the body. It can enhance proprioception as well as whole-body vibration therapy, improve the elasticity and mobility of local ligaments and tendons, promote the circulation of blood and lymphatic fluids around synovial joints, facilitate the secretion and flow of synovial fluid, and reduce joint capsule swelling and contracture [46]. The same beneficial effects also exist in the treatment of CLBP.

However, we still need to carefully explain the results of this study. Firstly, regarding the parameter settings of vibration therapy, some studies have reported that vibration close to the resonant frequency of the human body can cause damage to the spine [47] and that exposure to whole-body vibration in the work environment may lead to low back pain [48]. Kim et al. [49] also concluded that there is a very strong correlation between irregular vibration and musculoskeletal disorders, especially the occurrence of LBP. But this is not contradictory to the conclusion of this study. The therapeutic effect of vibration therapy is influenced by the frequency and amplitude of vibration. For the treatment of LBP with vibration therapy, how to choose the best vibration parameters is not clear, although irregular whole-body vibration can induce LBP. Secondly, the results of this study, although significant, do not appear to reach the levels of clinical relevance suggested by Maughan and Lewis [50]. According to their study, the following values represent the least clinically significant difference in CLBP: a mean difference of 2.4 in pain intensity on a scale of 0–10, a mean difference of 17 in ODI, and a mean difference of 5 in RMDQ. More large-sample, rigorously designed RCTs are needed to validate the efficacy of vibration therapy in patients with CLBP in the future.

Limitations of this paper: ① The amount of the literature included was limited, and the sample size included was small. ② The included studies varied widely in treatment methods and were not analyzed based on parameters such as vibration frequency and vibration amplitude, which may compromise the evaluation results. ③ There was a large degree of heterogeneity among the RCTs included in the combined analysis of pain intensity index scores and ODI in patients with CLBP. ④ Many of the RCTs included in this study lacked post-treatment follow-up and did not evaluate whether or not the efficacy was durable. ⑤ This study did not conduct a sensitivity analysis based on the risk of bias.

## Conclusion

The available evidence suggests that vibration therapy can alleviate pain and improve function in patients with CLBP. However, we still need to carefully interpret the results of this study, as the certainty of evidence was low, and the clinical relevance of the results is questionable. Further RCTs are needed in the future to ascertain this.

### Supplementary Information

**Additional file 1: ** Search strategy in PubMed.

**Additional file 2: ** Certainty of evidence assessment.

## Data Availability

The datasets used and analyzed during the current study are available from the corresponding author on reasonable request.

## Abbreviations

VT: Vibration therapy
LV: Local vibration
WBV: Whole-body vibration
RCTs: Randomized controlled trials
CLBP: Chronic low back pain
VAS: Visual analog scale
NRS: Numerical rating scale
ODI: Oswestry disability index
RMDQ: Roland–Morris disability questionnaire
SMD: Standardized mean difference
MD: Mean difference
SD: Standard deviation
CI: Confidence interval
NSAIDs: Nonsteroid anti-inflammatory drugs
