# Supplementary material for: Vibration therapy to improve pain and function in patients with chronic low back pain: a systematic review and meta-analysis
Source: J Orthop Surg Res. 2023 Sep 26;18:727. doi: 10.1186/s13018-023-04217-2 (PMC10523661; doi:10.1186/s13018-023-04217-2)
Supplement: Supplementary file 1 — Additional file 1: Search strategy in PubMed. [file 13018_2023_4217_MOESM1_ESM.docx]

**Additional file 1**

Search strategy in PubMed:

#1 Low Back Pain[MeSH Terms]

#2 Back Pain,Low[Title/Abstract] OR Back Pains,Low [Title/Abstract] OR Low Back Pains[Title/Abstract] OR Pain,Low Back [Title/Abstract] OR Pains,Low Back[Title/Abstract] OR Lumbago[Title/Abstract] OR Lower Back Pain[Title/Abstract] OR Back Pain,Lower[Title/Abstract] OR Back Pains,Lower[Title/Abstract] OR Lower Back Pains[Title/Abstract] OR Pain,Lower Back[Title/Abstract] OR Pains,Lower Back[Title/Abstract] OR Low Back Ache[Title/Abstract] OR Ache,Low Back[Title/Abstract] OR Aches,Low Back[Title/Abstract] OR Back Ache,Low[Title/Abstract] OR Back Aches,Low[Title/Abstract] OR Low Back Aches[Title/Abstract] OR Low Backache[Title/Abstract] OR Backache,Low[Title/Abstract] OR Backaches,Low[Title/Abstract] OR Low Backaches[Title/Abstract] OR Low Back Pain,Postural[Title/Abstract] OR Postural Low Back Pain[Title/Abstract] OR Low Back Pain,Posterior Compartment[Title/Abstract] OR Low Back Pain,Recurrent[Title/Abstract] OR Recurrent Low Back Pain[Title/Abstract] OR Low Back Pain,Mechanical[Title/Abstract] OR Mechanical Low Back Pain[Title/Abstract]

#3 #1OR#2

#4 Vibration[MeSH Terms] OR Vibrations[Title/Abstract] OR vibration[All Fields]

#5 randomized controlled trial[Publication Type] OR randomized controlled trials as topic[MeSH Terms] OR randomized controlled trial[All Fields] OR randomised controlled trial[All Fields] OR clinical trial[Publication Type] OR clinical trials as topic[MeSH Terms] OR clinical trial[All Fields]

#6 #3 AND #4 AND #5
