# Supplementary material for: Vibration therapy to improve pain and function in patients with chronic low back pain: a systematic review and meta-analysis
Source: J Orthop Surg Res. 2023 Sep 26;18:727. doi: 10.1186/s13018-023-04217-2 (PMC10523661; doi:10.1186/s13018-023-04217-2)
Supplement: Supplementary file 2 — Additional file 2: Certainty of evidence assessment. [file 13018_2023_4217_MOESM2_ESM.docx]

**Additional file 2**

**Table S1. Certainty of Evidence Assessment**

| Outcome | Study Design | Risk of bias | Inconsistency | Indirectness | Imprecision | Other considerations | No. of patients | | Absolute Effect(95%CI) | Certainty |
| --- | --- | --- | --- | --- | --- | --- | --- | --- | --- | --- |
|  |  |  |  |  |  |  | VT | Control |  |  |
| Pain intensity indicators | RCTs | Serious | Serious | Not serious | Not serious | None | 369 | 368 | SMD-0.71,(-1.02, -0.39) | Low |
| Pain intensity indicators in the WBV group | RCTs | Serious | Serious | Not serious | Not serious | None | 230 | 228 | SMD-0.49, (-0.79,-0.19) | Low |
| Pain intensity indicators in the LV group | RCTs | Serious | Serious | Not serious | Not serious | None | 139 | 140 | SMD-1.07,(-1.60,-0.53) | Low |
| Oswestry disability index | RCTs | Serious | Serious | Not serious | Not serious | None | 197 | 193 | MD-4.24,(-8.10, -0.38) | Low |
| Oswestry disability index in the WBV group | RCTs | Serious | Serious | Not serious | Not serious | None | 128 | 124 | MD-3.30,(-5.76,-0.83) | Low |
| Oswestry disability index in the LV group | RCTs | Serious | Serious | Not serious | Not serious | None | 69 | 69 | MD-5.78, (-16.23,4.66) | Low |
| Roland-Morris disability questionnaire | RCTs | Serious | Not serious | Not serious | Serious | None | 71 | 68 | MD-2.21,(-3.41, -1.01) | Low |
